# Supplementary material for: Dynamical Behavior of Two Interacting Double Quantum Dots in 2D Materials for Feasibility of Controlled-NOT Operation
Source: Nanomaterials (Basel). 2022 Oct 13;12(20):3599. doi: 10.3390/nano12203599 (PMC9610695; doi:10.3390/nano12203599)
Supplement: Supplementary file 1 [file nanomaterials-12-03599-s001.zip › nanomaterials-1962552-supplementary-conversion/Supplementary_Proofread/Supplementary_Proofread.pdf]

Dynamical Behavior of Two Interacting Double Quantum Dots in 2D Materials for Feasibility of Controlled-NOT Operation: Supplementary

Aniwat Kesorn[1],
Rutchapon Hunkao[1],
Kritsanu Tivakornsasithorn[1],
Asawin Sinsarp[1],
Worasak Sukkabot[2],
Sujin Suwanna[1*]

[1]Optical and Quantum Physics Laboratory, Department of Physics, Faculty of Science, Mahidol University, Bangkok, 10400, Thailand

[2]Department of Physics, Faculty of Science, Ubon Ratchathani University, Ubon Ratchathani, 34190, Thailand

Correspondence: sujin.suw@mahidol.ac.th

S1 Matrix model

As mentioned in the main article, the Hamiltonian H_{2q} of the two DQDs with the Coulomb interaction of electrons between the DQDs is written as

$$H_{2q} = \begin{pmatrix} -\frac{1}{2}(\varepsilon_l + \varepsilon_r) + J_2 & -\frac{1}{2}\Delta_r & -\frac{1}{2}\Delta_l & 0 \\ -\frac{1}{2}\Delta_r & -\frac{1}{2}(\varepsilon_l - \varepsilon_r) + J_3 & 0 & -\frac{1}{2}\Delta_l \\ -\frac{1}{2}\Delta_l & 0 & -\frac{1}{2}(-\varepsilon_l + \varepsilon_r) + J_1 & -\frac{1}{2}\Delta_r \\ 0 & -\frac{1}{2}\Delta_l & -\frac{1}{2}\Delta_r & -\frac{1}{2}(-\varepsilon_l - \varepsilon_r) + J_2 \end{pmatrix} \tag{S1}$$

The Hamiltonian in equation (S1) can be extracted for the subsystems, whose Hamiltonians for the left and right DQDs are respectively given by

$$\begin{aligned} H_l &= -\frac{1}{2}\left(\varepsilon_l \sigma_z + \Delta_l \sigma_x\right) + J_2\left(\langle R|0\rangle_r \langle 0|_r R\rangle\right) |0\rangle_l \langle 0|_l \\ &\quad + J_3\left(\langle R|1\rangle_r \langle 1|_r R\rangle\right) |0\rangle_l \langle 0|_l + J_1\left(\langle R|0\rangle_r \langle 0|_r R\rangle\right) |1\rangle_l \langle 1|_l \\ &\quad + J_2\left(\langle R|1\rangle_r \langle 1|_r R\rangle\right) |1\rangle_l \langle 1|_l \end{aligned} \tag{S2}$$

$$\begin{aligned} H_r &= -\frac{1}{2}\left(\varepsilon_r \sigma_z + \Delta_r \sigma_x\right) + J_2\left(\langle L|0\rangle_l \langle 0|_l L\rangle\right) |0\rangle_r \langle 0|_r \\ &\quad + J_3\left(\langle L|1\rangle_l \langle 1|_l L\rangle\right) |0\rangle_r \langle 0|_r + J_1\left(\langle L|0\rangle_l \langle 0|_l L\rangle\right) |1\rangle_r \langle 1|_r \\ &\quad + J_2\left(\langle L|1\rangle_l \langle 1|_l L\rangle\right) |1\rangle_r \langle 1|_r \end{aligned} \tag{S3}$$

If the state can be written as a product of the subsystem, the solution of H_{2q} in equation (S1) and that from the product solutions of H_l in equation (S2) and H_r in equation (S3) are the same, as shown in Figure S1.

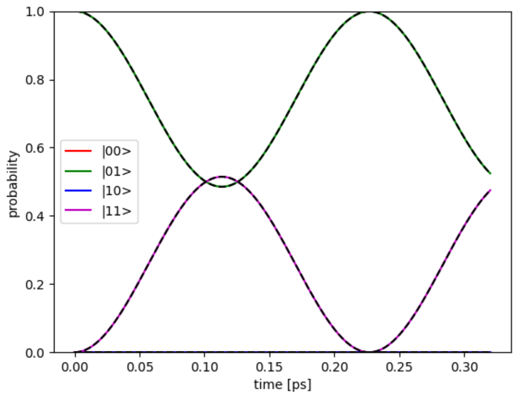

Figure S1: The dynamics of states of two DQDs with the inter-DQD Coulomb interaction for the initial state $|01\rangle$, the QD base length $b = 2.0$ nm, the inter-QD distance $d = 3.0$ nm. The two DQDs are separated by the inter-DQD distance $a = 8.0$ nm. The solid and dash lines show the results from equation (S1) and from equations (S2) and (S3), respectively.

S2 Supercell size

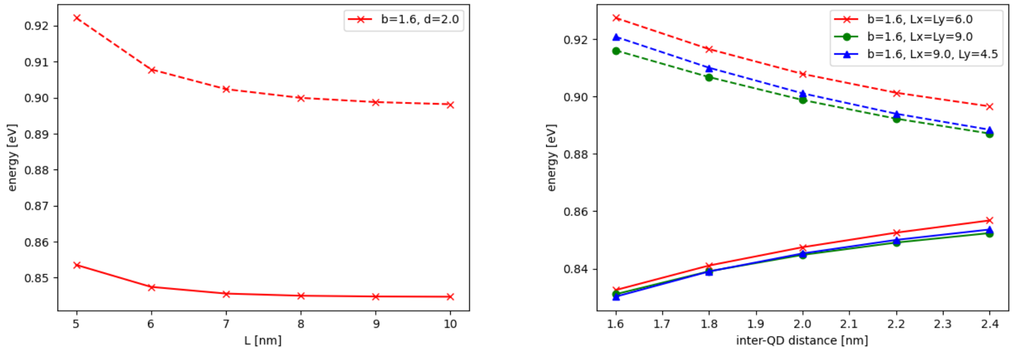

(a)

(b)

Figure S2: (a) The DQD energy as a function of the supercell length $L_x = L_y = L$. (b) The DQD energy as a function of the inter-QD distance d with different supercell length L_x and L_y. The solid and dash lines denote the bonding and anti-bonding energies, respectively.

The geometrical parameters, the potential and the spatial-dependent effective mass of MoS_2 are used in simulation. The height of the QDs is though they can be varied as desired scaled by $h = b/2$. In Figure S2, the solid and dash lines represent the bonding and anti-bonding state energies. In Figure S2a, the DQD energies converge when the supercell is large. In Figure S2b, we compare energies the different supercell lengths with $L_x = L_y = 6.0$ nm, $L_x = L_y = 9.0$ nm and $L_x = 9.0$ nm, $L_y = 4.5$ nm. The supercell with $L_x = L_y = 9.0$ nm (denoted by circle green lines) is large enough for convergence. The calculation with $L_x = 9.0$ nm, $L_y = 4.5$ nm (or blue lines) have accuracy close to that the green lines, but the calculation time is shorter by about four times. Hence, we have decided to use the supercell of dimensions $L_x = 9.0$ nm, $L_y = 4.5$ nm for calculating the dynamics of states.

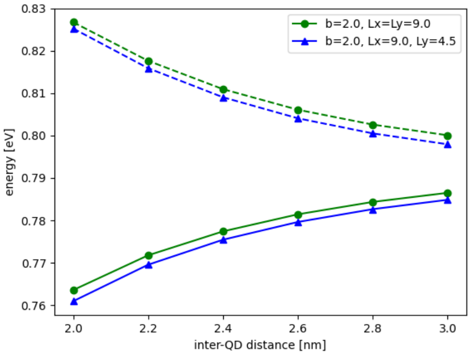

Figure S3: The DQD energy as a function of the inter-QD distance d with different supercell lengths L_x and L_y. The solid and dash lines are bonding and anti-bonding energies, respectively.

S3 Fitting energy of the DQD

S3.1 Energy of 1D double quantum wells with WKB approximation

Considering the 1D double quantum wells as depicted by the diagram in Figure S4. Two finite potential wells with a potential V_0, each of width b, are separated by distance d. The two wells are embedded in the infinite well of length L. We are interested in the energies $E < V_0$ for bound of state of an electron in the wells. Using the WKB approximation, the quantization condition of energy for symmetrical double wells is $tan(\theta) = \pm 2e^{\phi}$, see J. D. Griffiths *Introduction to Quantum Mechanics* [54] in Chapter 8 for more details. The variables θ and ϕ are calculated by the integral following

$$\theta = \frac{1}{\hbar}\int_{x_1}^{x_2} p(x)\,dx = \frac{1}{\hbar}\int_{d/2}^{d/2+b} \sqrt{2mE}\,dx = \frac{b}{\hbar}\sqrt{2mE}, \tag{S4}$$

$$\phi = \frac{1}{\hbar}\int_{-x_1}^{x_1} |p(x')|\,dx' = \frac{1}{\hbar}\int_{-d/2}^{d/2} \sqrt{2m(V_0 - E)}\,dx' = k\sqrt{V_0 - E}, \tag{S5}$$

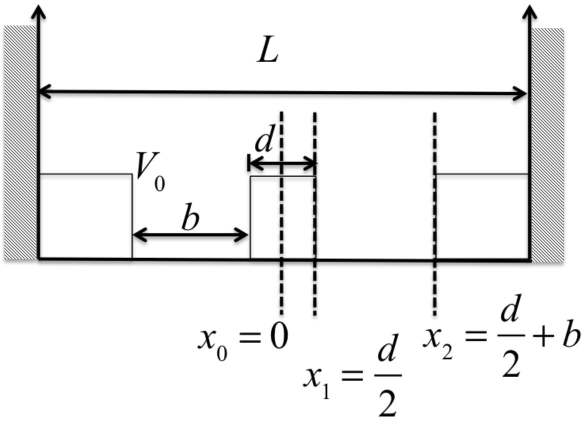

Figure S4: Diagram of 1D double quantum wells.

where $k = \sqrt{2m}d/\hbar$. In case of high and/or broad central barrier (V_0 and d), ϕ is large and e^{ϕ} becomes even larger. It means that θ is close to a half integer multiple of π. Then, the quantization condition can be approximated as

$$\theta \approx (n + \frac{1}{2})\pi \mp \frac{1}{2}e^{-\phi}. \tag{S6}$$

Using equations (S4) and (S6), the electron energy can be approximated as

$$E = (n + \frac{1}{2})^2 \frac{\pi^2 \hbar^2}{2mb^2} + \frac{\hbar^2}{8mb^2} e^{-2\phi} \mp (n + \frac{1}{2}) \frac{\pi \hbar^2}{2mb^2} e^{-\phi}. \tag{S7}$$

The energy level n splits into two values. We are interested in the first lowest level, and denote this splitting as the bonding E^b and anti-bonding E^{ab} energies. Defining the parameter $\beta = \hbar^2/(2mb^2)$, the splitting energies and the variable ϕ in equation (S5) are given by

$$E^b = (n + \frac{1}{2})^2 \pi^2 \beta + \frac{1}{4} \beta e^{-2\phi^b} - (n + \frac{1}{2}) \pi \beta e^{-\phi^b} \tag{S8}$$

$$E^{ab} = (n + \frac{1}{2})^2 \pi^2 \beta + \frac{1}{4} \beta e^{-2\phi^{ab}} + (n + \frac{1}{2}) \pi \beta e^{-\phi^{ab}} \tag{S9}$$

$$\phi^b = k\sqrt{V_0 - E^b} \tag{S10}$$

$$\phi^{ab} = k\sqrt{V_0 - E^{ab}}. \tag{S11}$$

Therefore, the energy splitting of the bonding and anti-bonding states is $\Delta = E^{ab} - E^b$. Solving these equations to find close forms for E and ϕ is difficult because they nonlinearly depend on other parameters. For some symmetrical double wells, using the WKB without additional approximation of the quantization condition, the numerical solutions of the coupling equation for the energy splitting are demonstrated and compared to the variational principle method in Ref. [53] In the coupling equations above, for the approximation of the splitting energy, we assume that the bonding and anti-bonding energies are symmetrical splitting around mean energy $\tilde{E}$. Then, the bonding and anti-bonding energies can be written as

$$E^b = \tilde{E} - \epsilon \ \text{ and } \ E^{ab} = \tilde{E} + \epsilon, \tag{S12}$$

so that $\Delta = E^{ab} - E^b = 2\epsilon$. Then, the variables ϕ^b and ϕ^{ab} are

$$\phi^{b,ab} = k\sqrt{V_0 - E^{b,ab}} = k\sqrt{V_0 - \tilde{E} \pm \epsilon}. \tag{S13}$$

Then, approximating equation (S13) yields

$$\phi^b \approx k\sqrt{V_0 - \tilde{E}} \left(1 + \frac{\epsilon}{2(V_0 - \tilde{E})}\right), \tag{S14}$$

$$\phi^{ab} \approx k\sqrt{V_0 - \tilde{E}} \left(1 - \frac{\epsilon}{2(V_0 - \tilde{E})}\right). \tag{S15}$$

If we define $\tilde{\phi} = k\sqrt{V_0 - \tilde{E}}$ and $\delta = (\epsilon k) \, / \left(2\sqrt{V_0 - \tilde{E}}\right)$, then we can write $\phi^b = \tilde{\phi} + \delta$ and $\phi^{ab} = \tilde{\phi} - \delta$. Using equation (S12), the relation of the parameter δ and splitting energy Δ is obtained via

$$\delta = \frac{k}{\sqrt{V_0 - \tilde{E}}} \frac{\Delta}{4}. \tag{S16}$$

Considering, the splitting energy in terms of the parameters $\tilde{\phi}$ and δ

$$\begin{aligned}
\Delta &= E^{ab} - E^b \\
&\approx \frac{\beta}{4} e^{-2\tilde{\phi}} \left(e^{2\delta} - e^{-2\delta}\right) + (n + \frac{1}{2}) \pi \beta e^{-\tilde{\phi}} \left(e^{\delta} + e^{-\delta}\right) \\
&= \frac{\beta}{4} e^{-2\tilde{\phi}} (4\delta) + (n + \frac{1}{2}) \pi \beta e^{-\tilde{\phi}} (2) \\
&= \frac{\beta}{4} e^{-2\tilde{\phi}} \left(\frac{k\Delta}{\sqrt{V_0 - \tilde{E}}}\right) + (n + \frac{1}{2}) 2\pi \beta e^{-\tilde{\phi}} \\
&= \frac{(n + \frac{1}{2}) 2\pi \beta e^{-k\sqrt{V_0 - \tilde{E}}}}{1 - \frac{\beta/4}{\sqrt{V_0 - \tilde{E}}} k e^{-2k\sqrt{V_0 - \tilde{E}}}}
\end{aligned} \tag{S17}$$

The term $1 - \frac{\beta/4}{\sqrt{V_0 - \tilde{E}}} k e^{-2k\sqrt{V_0 - \tilde{E}}}$ must be positive because the splitting energy Δ is positive. Therefore, $\frac{\beta/4}{\sqrt{V_0 - \tilde{E}}} k e^{-2k\sqrt{V_0 - \tilde{E}}} \ll 1$, where the variable k is proportional to the distance d. This term is zero

when $d = 0$, increases slightly, then rapidly decays to zero as d increases. Therefore, the denominator of equation (S17) is close to one when the ratio of $\beta/(4\sqrt{V_0 - \tilde{E}})$ is small. In the asymptotic limit of a vary large d, the two wells are considered independently, so the bonding and anti-bonding energies are close to the energy of one finite well, and the splitting tends to zero. The wavefunction of such bonding state is similar to that of one well with a half amplitude placing in two wells. Also, when there is no energy splitting for very large distance d, an electron is in one well entirely (with two wells having degenerate energy), or it is in the bonding state or anti-bonding state with half amplitudes. In equation (S17), the splitting energy is assumed symmetrical around mean the energy $\tilde{E}$ which will be considered as the energy in the asymptotic limit. Therefore, the term $\sqrt{V_0 - \tilde{E}}$ can be considered as a function depending on parameters of a single quantum well.

Equation (S17) a motivation to define the fitting function of the 2D DQD energy gap in the main article as the maximum energy gap multiplied by the exponential decay as a function of the separating distance of QDs $(d - b)$. The decay rate depends on the parameters of a quantum dot. That is, $\Delta = \Delta_{max}(V, b)e^{-\alpha(V,b)(d-b)}$.

S3.2 Fitting energy of the DQD

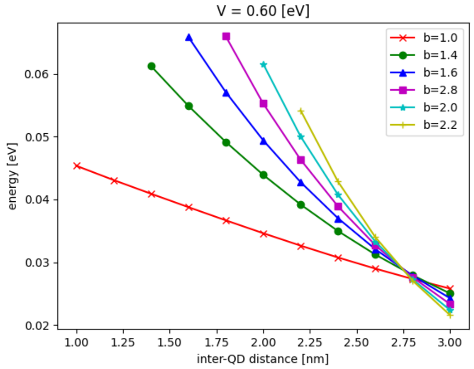

(a)

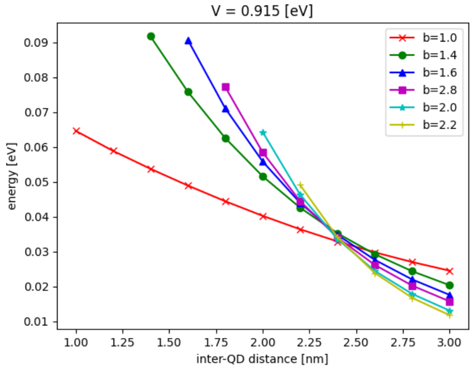

(b)

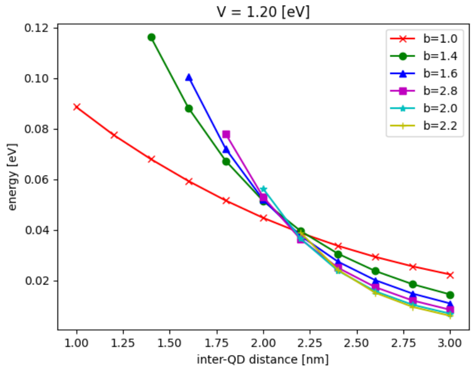

(c)

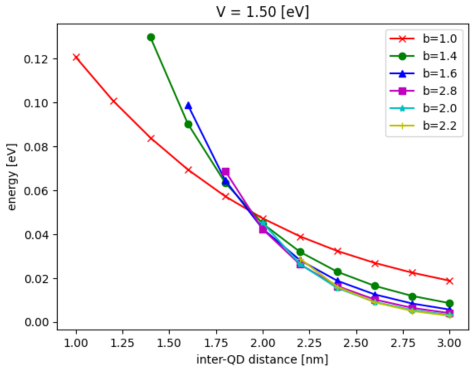

(d)

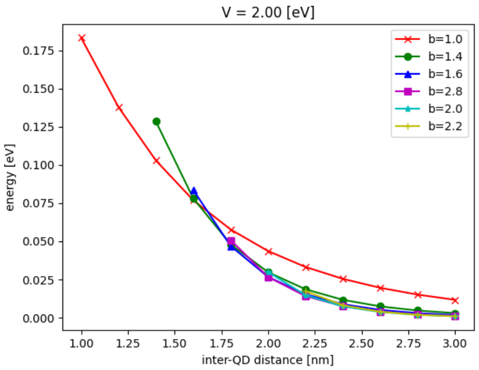

(e)

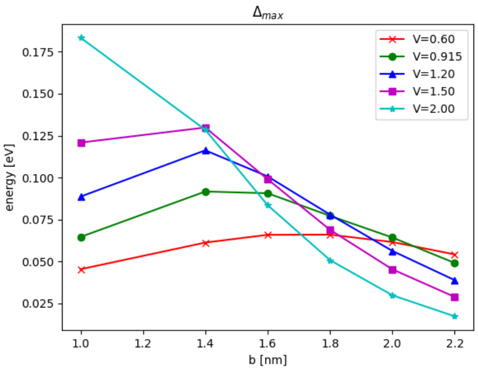

(f)

Figure S5: The energy gaps between bonding and anti-bonding states of an electron as a function of the inter-QD distance d which the different electronic potential (a) $V = 0.60$ eV, (b) $V = 0.915$ eV, (c) $V = 1.20$ eV, (d) $V = 1.50$ eV, (e) $V = 2.00$ eV the color curves represent the different QD base lengths; (f) the maximum energy gaps as a function of the QD base length b which different potential values.

	V				
b	0.600	0.915	1.200	1.500	2.000
1.0	0.278	0.481	0.689	0.935	1.401
1.4	0.560	0.949	1.322	1.781	2.382
1.6	0.717	1.184	1.608	2.058	2.797
1.8	0.873	1.341	1.874	2.371	3.158
2.0	1.019	1.601	2.122	2.652	3.465
2.2	1.157	1.792	2.350	2.900	3.726

Table S1: The fitting of the exponential component α as a function of V and b.

	V				
b	0.600	0.915	1.200	1.500	2.000
1.0	0.99866	0.99954	0.99979	0.99986	0.99912
1.4	0.99994	0.99983	0.99941	0.99933	0.99960
1.6	0.99995	0.99965	0.99957	0.99969	0.99981
1.8	0.99987	0.99950	0.99979	0.99985	0.99989
2.0	0.99987	0.99986	0.99990	0.99991	0.99993
2.2	0.99991	0.99993	0.99994	0.99994	0.99995

Table S2: R^2 of the fitting of exponential component α.

V	$m_1(V)$	$c_1(V)$
0.600	0.7400	-0.4460
0.915	1.0881	-0.5888
1.200	1.3837	-0.6454
1.500	1.6193	-0.5826
2.000	1.9367	-0.4064

Table S3: The fitting of parameters m_1 and c_1 as a function of V.

S4 Initial state of DQD under electric field strength

In this section, the suitable initial preparations are given for some examples by varying the electric field strength. The ideal qubit states $|0\rangle$ and $|1\rangle$ are defined by a linear combination of two bonding and anti-bonding states of the DQD without the electric field strength. The DQDs are prepared in the position-dependent eigenstates of the Hamiltonian with the electric field, which can define the initial state $|0\rangle$ or $|1\rangle$. Such initial are verified by the projection onto the ideal qubit states. Suppose we need to prepare the initial state such as $|0\rangle$ in Figures S6 and S7. If the applied external electric field is too weak or too strong, then the initial state is prepared with less accuracy.

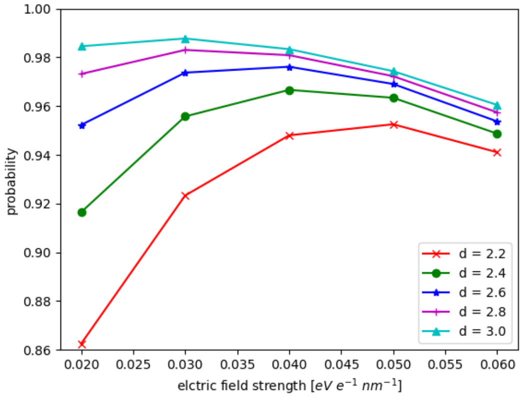

Figure S6: The probability of preparing the initial state in $|0\rangle$ when the DQD is under the applied electric field strength varying in strength. The potential parameter $V = 0.915$ eV (MoS_2), the QD base $b = 1.8$ nm and the inter-QD distance d are used in calculating the color lines.

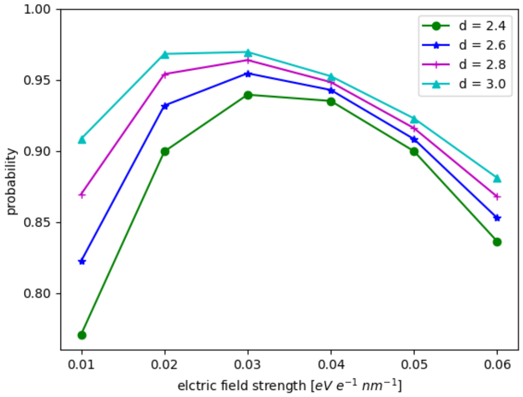

Figure S7: The probability of preparing the initial state in $|0\rangle$ when the DQD is under the applied electric field strength varying in strength. The potential parameter $V = 0.60$ eV, the QD base $b = 2.0$ nm and the inter-QD distance d are used in calculating the color lines.

S5 Electronic potential and matrix models with varying time steps.

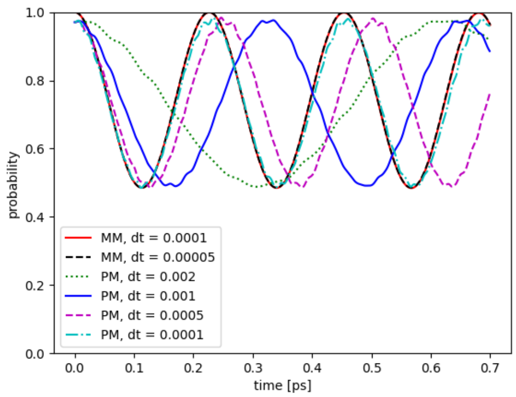

Figure S8: The dynamics of states of two DQDs with the inter-DQD Coulomb interaction with the initial state $|01\rangle$, the QD base length $b = 2.0$ nm, the inter-QD distance $d = 3.0$ nm and the two DQDs are separated by the inter-DQD distance $a = 8.0$ nm. The dynamics of the state $|01\rangle$ are shown as a function of time for the electronic potential model (PM) and the matrix model (MM). The time step (dt) of the calculation is varied in the different curves.

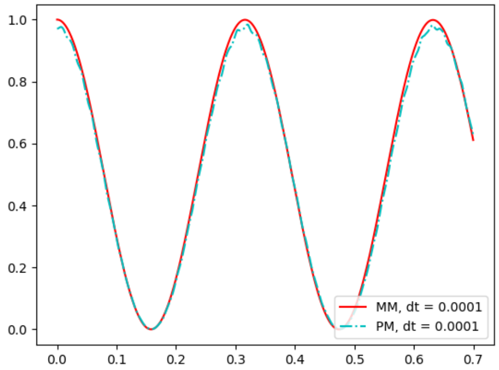

Figure S9: The dynamics of the state $|01\rangle$ of the the two DQDs without the inter-DQD Coulomb interaction.

In Figure S8, the dynamics of the state $|01\rangle$ from the electronic potential model (PM) are compared with those from the matrix model (MM). The time step (dt) of the calculation is varied. The electronic potential model is calculated by the Crank-Nicolson (CN) method and the matrix mode is calculated by the fourth order Runge–Kutta (RK) method. For a larger time step, the time evolution with the RK method in the matrix model does not conserve the norm of the states, but that with the CN method in the electronic potential model does. However, in the electronic potential model, a larger time step make the errors accumulate more as the calculation time increases. For the smaller time step, the results of the two models are nearly identical.

In the case of two DQDs without the inter-DQD interaction, in the matrix model, we know exactly the time period for the dynamics of state to again return to the initial state; that period is given by $t_{2\pi} = 2\pi\hbar/\Delta = 0.316$ ps, where Δ is the energy gap. The numerical results are good agreement as evident in Figure S9.

S6 CNOT operation efficiency ΔP

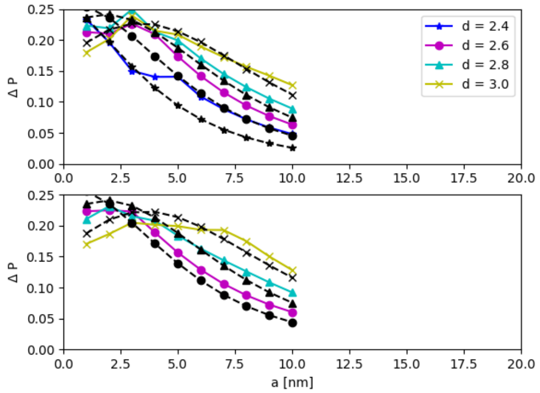

Figure S10: The CNOT operation efficiency ΔP as a function of the inter-DQD distance a, the QD base lengths $b = 2.0$ nm and $b = 2.2$ nm are shown in the top and bottom panels, respectively. The electronic potential parameter is $V = 0.60$ eV. The results from different the inter-QD distance d are denoted by different symbols. The solid and dash lines are from the electronic potential and matrix models, respectively.

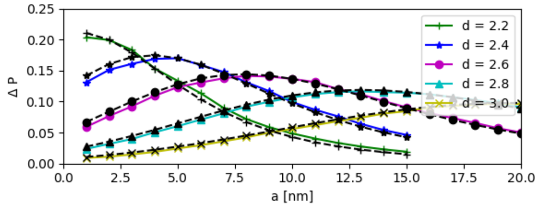

Figure S11: The CNOT operation efficiency ΔP as a function of the inter-DQD distance a, the QD base lengths $b = 1.8$ nm. The electronic potential parameter is $V = 1.50$ eV. The results from different the inter-QD distance d are denoted by different symbols. The solid and dash lines are from the electronic potential and matrix models, respectively.

S7 The dynamics of state with hypothetical parameters

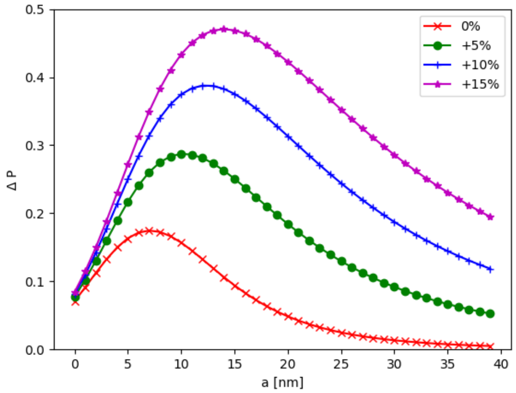

Figure S12: The CNOT operation efficiency ΔP as a function of the inter-DQD distance a, the QD base length $b = 2.0$ nm and the inter-QD distance $d = 3.0$ nm. This structure gives the parameters $\Delta_l = \Delta_r = 0.013083$ eV in matrix model. We use $\varepsilon_l = 0.0$ eV, $\varepsilon_r = 2.0$ eV. The inter-qubit interaction J_1 is artificially increased by 5%, 10% and 15% and the others (J_2 and J_3) are not changed. Then, the ΔP increases by almost 2 - 3 folds.

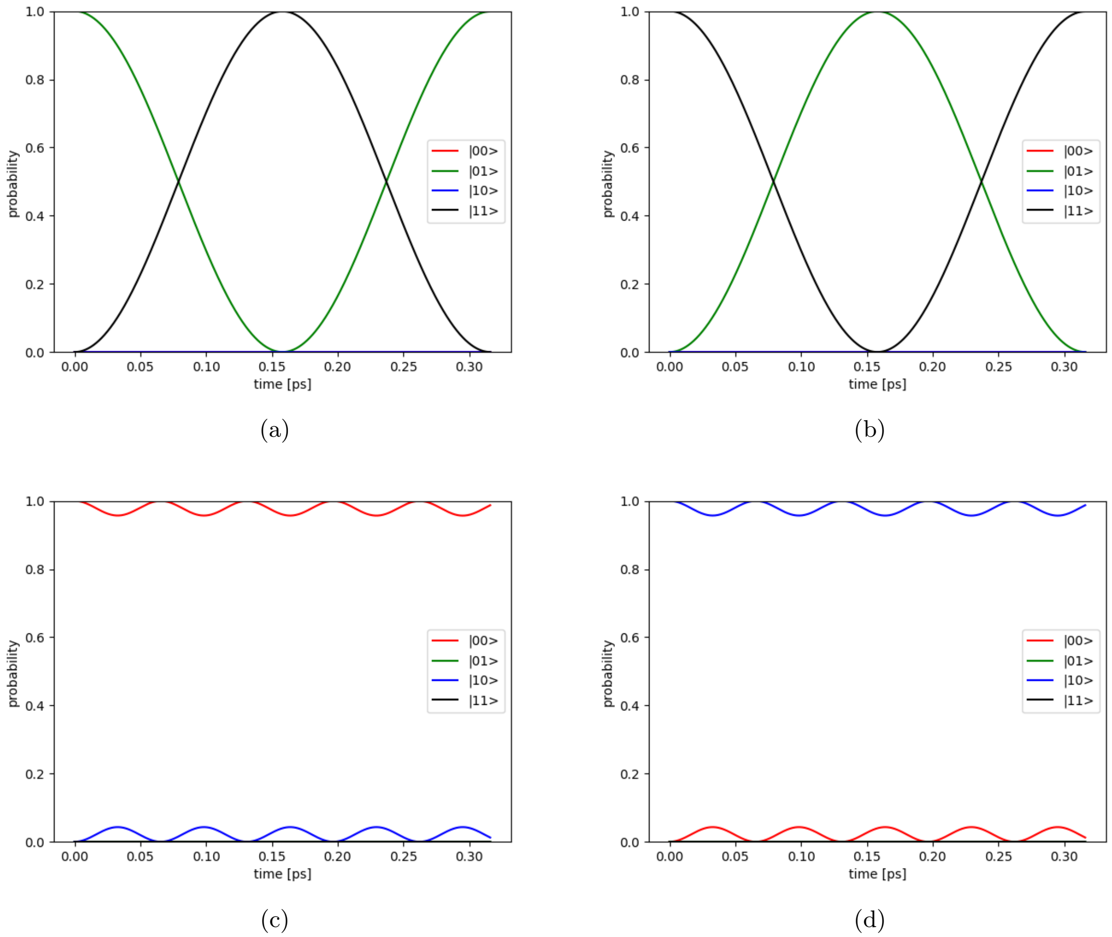

Figure S13: The dynamics of states in the matrix model are simulated with hypothetical parameters for the CNOT operation. The probability as a function of time in two qubit states are shown for the initial states (a) $|01\rangle$, (b) $|11\rangle$, (c) $|00\rangle$ and (d) $|10\rangle$.

The DQD structure has the QD base length $b = 2.0$ nm, the inter-QD distance $d = 3.0$ nm, and inter-DQD distance $a = 8.0$ nm. The parameters of the matrix model can be extracted from the electronic potential model as $\Delta_l = \Delta_r = 0.013083$ eV. The inter-qubit interactions are $J_1 = 0.102854$ eV, $J_2 = 0.084704$ eV and $J_3 = 0.071998$ eV calculating from the model of distance for this structure. We use $\varepsilon_l = 0.0$ eV, $\varepsilon_r = 2.0$ eV for the CNOT simulation. The dynamics of state for the initial state $|01\rangle$ is given in the Figure S1.

For a hypothetical set of parameters $\{J_1,\ J_2,\ J_3\}$ such as small $J_2 \approx J_3$ and very strong J_1, we choose the parameters above as $J_2 = J_3 = 0.071998$ eV and $J_1 = 1.3J_1$. In CNOT simulation for the right qubit controlling the left qubit, the dynamics of state are illustrated in Figure S13 for different initial states. We see that if the right qubit is in the state $|1\rangle_r$ then the left qubit can be flipped under the operation. On the other hand, if the right qubit is in the state $|0\rangle_r$, then the left qubit remains almost in the initial state, satisfying the CNOT operation.
