# Supplementary figures and images for: Dynamical Behavior of Two Interacting Double Quantum Dots in 2D Materials for Feasibility of Controlled-NOT Operation
Source: Nanomaterials (Basel). 2022 Oct 13;12(20):3599. doi: 10.3390/nano12203599 (PMC9610695; doi:10.3390/nano12203599)

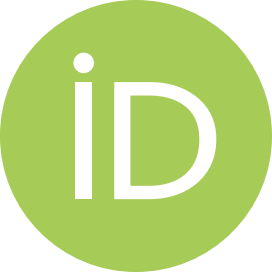

Supplement: Supplementary file 1 [file nanomaterials-12-03599-s001.zip › nanomaterials-1962552-supplementary-conversion/Supplementary_Proofread/Definitions/logo-orcid.pdf]

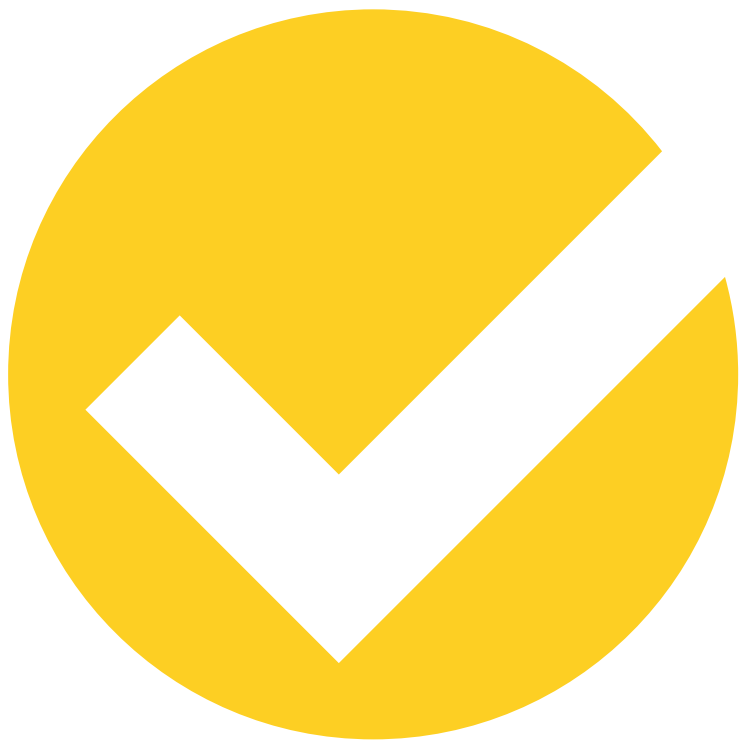

check for  
updates

Supplement: Supplementary file 1 [file nanomaterials-12-03599-s001.zip › nanomaterials-1962552-supplementary-conversion/Supplementary_Proofread/Definitions/logo-updates.pdf]

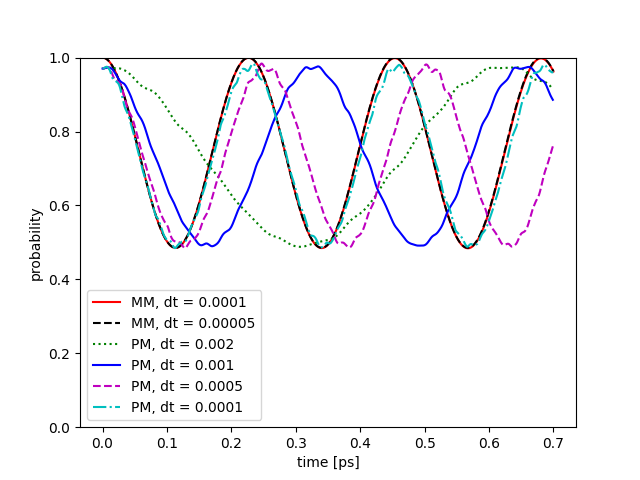

Supplement: Supplementary file 1 [file nanomaterials-12-03599-s001.zip › nanomaterials-1962552-supplementary-conversion/Supplementary_Proofread/figure_sup/dynamic_01_dt_2model.png]

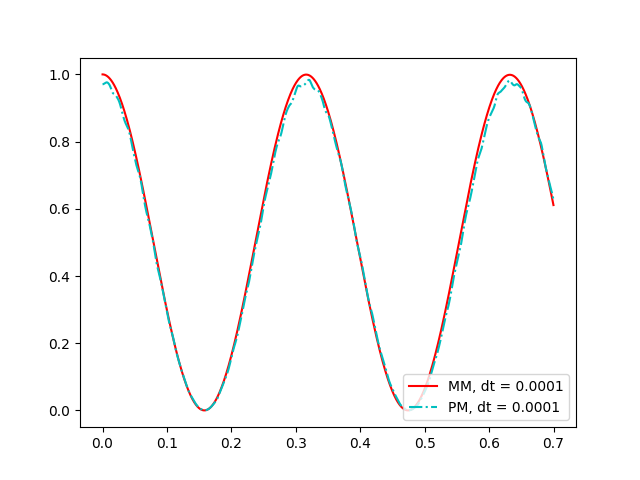

Supplement: Supplementary file 1 [file nanomaterials-12-03599-s001.zip › nanomaterials-1962552-supplementary-conversion/Supplementary_Proofread/figure_sup/dynamic_01_noninter_2model.png]

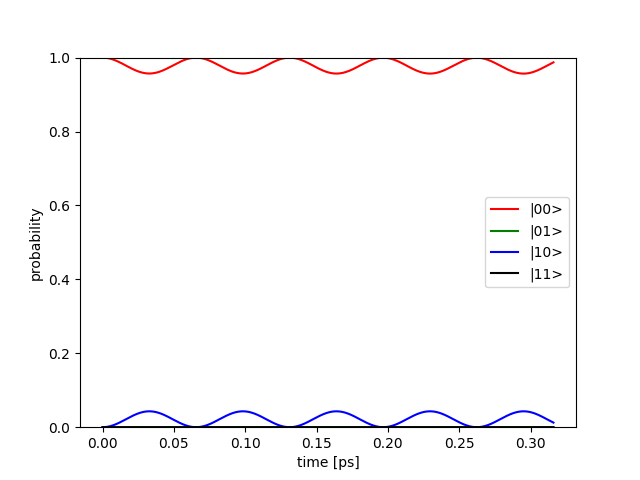

Supplement: Supplementary file 1 [file nanomaterials-12-03599-s001.zip › nanomaterials-1962552-supplementary-conversion/Supplementary_Proofread/figure_sup/dy_in00_ficpara.png]

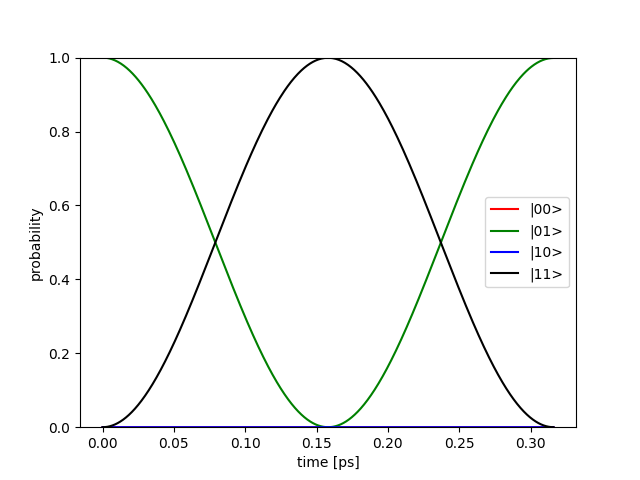

Supplement: Supplementary file 1 [file nanomaterials-12-03599-s001.zip › nanomaterials-1962552-supplementary-conversion/Supplementary_Proofread/figure_sup/dy_in01_ficpara.png]

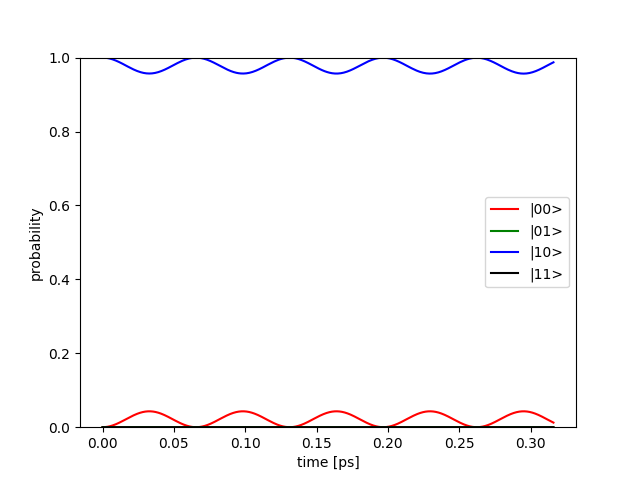

Supplement: Supplementary file 1 [file nanomaterials-12-03599-s001.zip › nanomaterials-1962552-supplementary-conversion/Supplementary_Proofread/figure_sup/dy_in10_ficpara.png]

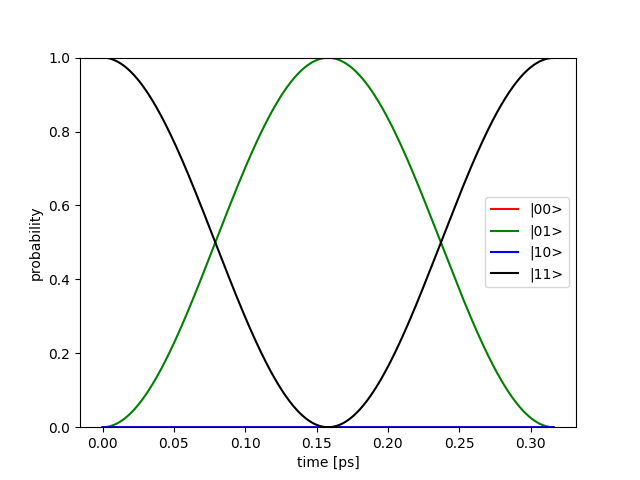

Supplement: Supplementary file 1 [file nanomaterials-12-03599-s001.zip › nanomaterials-1962552-supplementary-conversion/Supplementary_Proofread/figure_sup/dy_in11_ficpara.png]

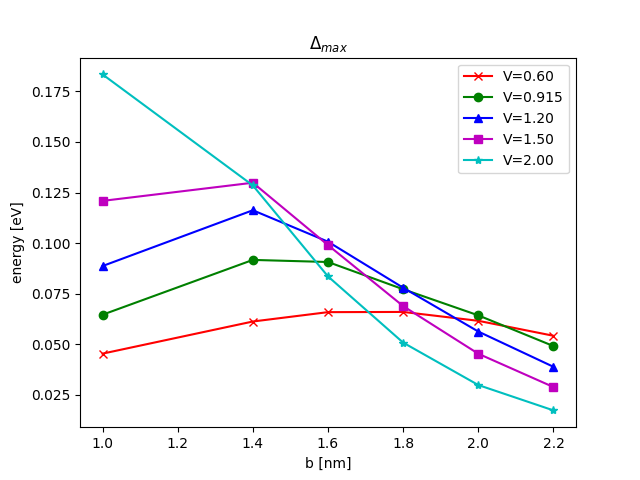

Supplement: Supplementary file 1 [file nanomaterials-12-03599-s001.zip › nanomaterials-1962552-supplementary-conversion/Supplementary_Proofread/figure_sup/Eg_dqd_max.png]

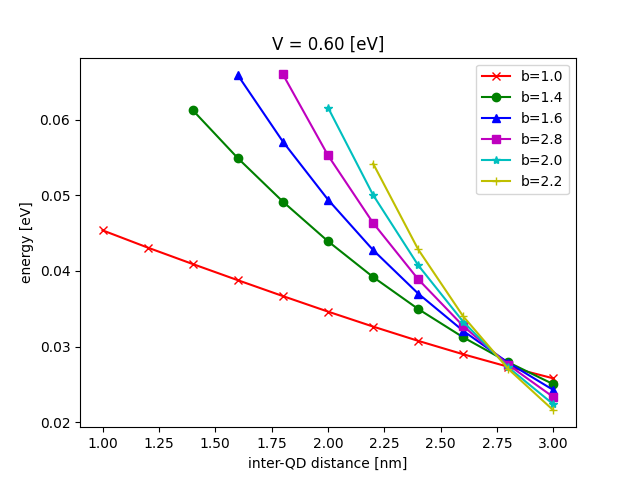

Supplement: Supplementary file 1 [file nanomaterials-12-03599-s001.zip › nanomaterials-1962552-supplementary-conversion/Supplementary_Proofread/figure_sup/Eg_dqd_V0d6.png]

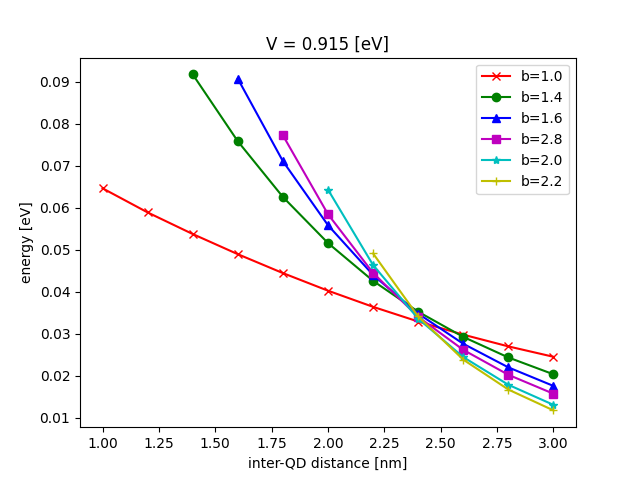

Supplement: Supplementary file 1 [file nanomaterials-12-03599-s001.zip › nanomaterials-1962552-supplementary-conversion/Supplementary_Proofread/figure_sup/Eg_dqd_V0d915_sup.png]

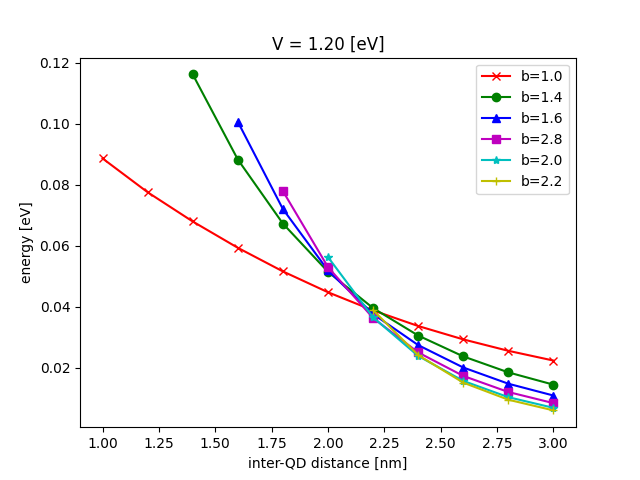

Supplement: Supplementary file 1 [file nanomaterials-12-03599-s001.zip › nanomaterials-1962552-supplementary-conversion/Supplementary_Proofread/figure_sup/Eg_dqd_V1d2.png]

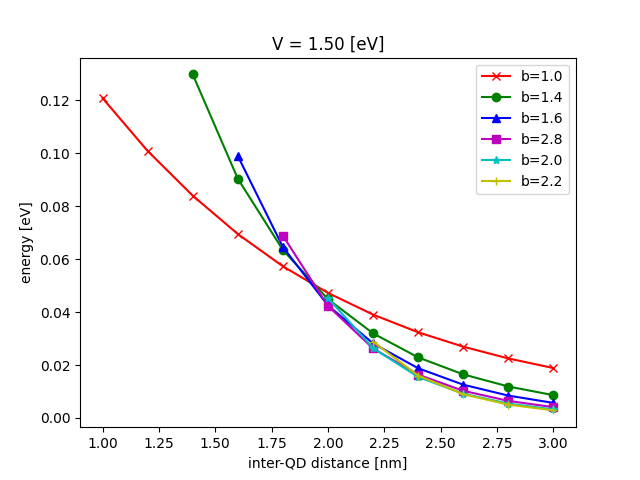

Supplement: Supplementary file 1 [file nanomaterials-12-03599-s001.zip › nanomaterials-1962552-supplementary-conversion/Supplementary_Proofread/figure_sup/Eg_dqd_V1d5.png]

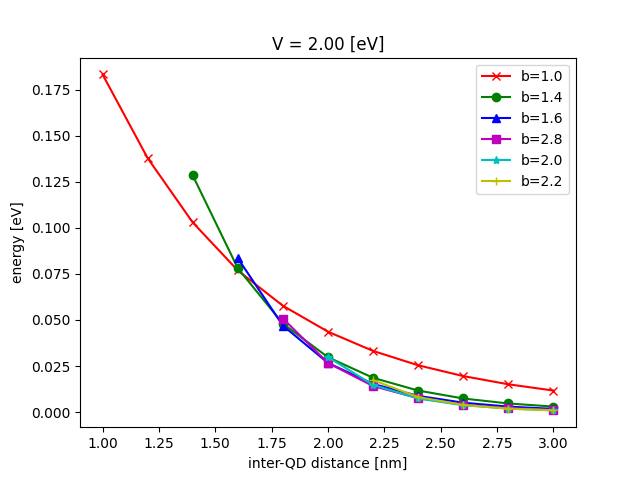

Supplement: Supplementary file 1 [file nanomaterials-12-03599-s001.zip › nanomaterials-1962552-supplementary-conversion/Supplementary_Proofread/figure_sup/Eg_dqd_V2d0.png]

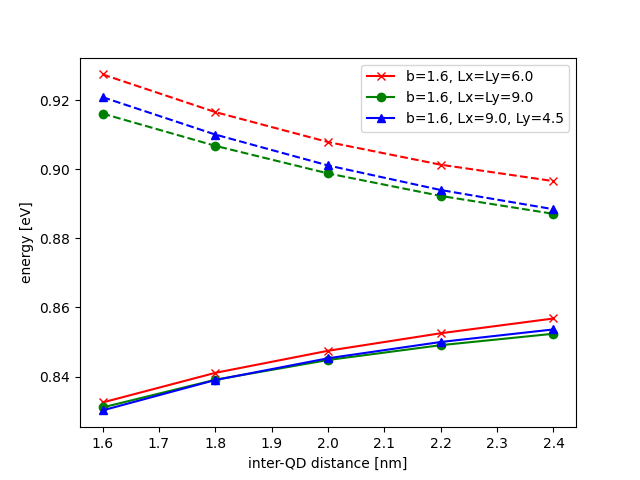

Supplement: Supplementary file 1 [file nanomaterials-12-03599-s001.zip › nanomaterials-1962552-supplementary-conversion/Supplementary_Proofread/figure_sup/En_dqd_Lcompare.png]

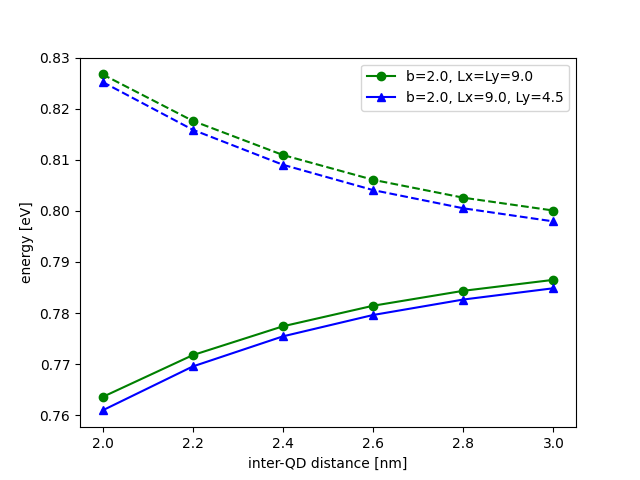

Supplement: Supplementary file 1 [file nanomaterials-12-03599-s001.zip › nanomaterials-1962552-supplementary-conversion/Supplementary_Proofread/figure_sup/En_dqd_Lcompare_b2d0.png]

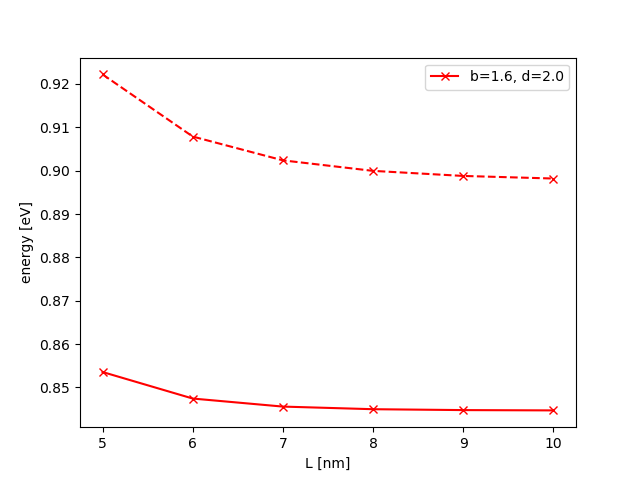

Supplement: Supplementary file 1 [file nanomaterials-12-03599-s001.zip › nanomaterials-1962552-supplementary-conversion/Supplementary_Proofread/figure_sup/En_dqd_Lconverge.png]

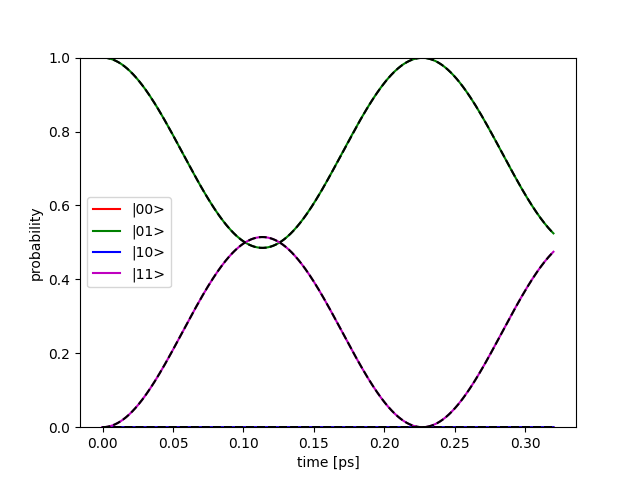

Supplement: Supplementary file 1 [file nanomaterials-12-03599-s001.zip › nanomaterials-1962552-supplementary-conversion/Supplementary_Proofread/figure_sup/mm1_mm2_compare.png]

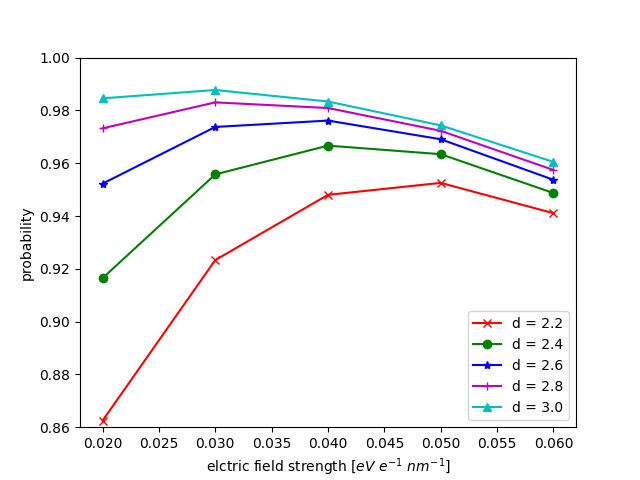

Supplement: Supplementary file 1 [file nanomaterials-12-03599-s001.zip › nanomaterials-1962552-supplementary-conversion/Supplementary_Proofread/figure_sup/P0_Ef_b1d8_V0d915.png]

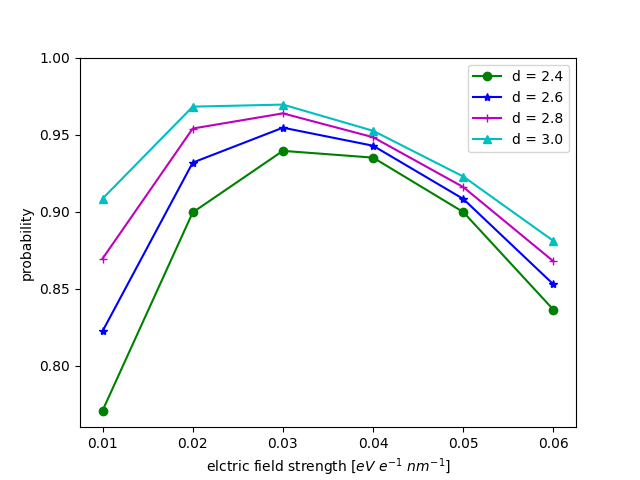

Supplement: Supplementary file 1 [file nanomaterials-12-03599-s001.zip › nanomaterials-1962552-supplementary-conversion/Supplementary_Proofread/figure_sup/P0_Ef_b2d0_V0d6.png]

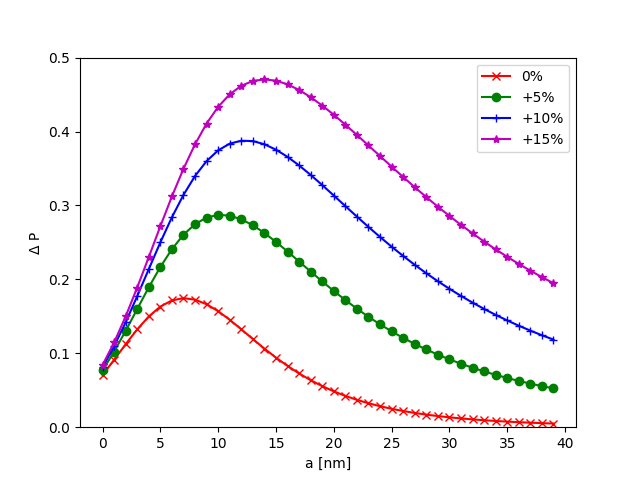

Supplement: Supplementary file 1 [file nanomaterials-12-03599-s001.zip › nanomaterials-1962552-supplementary-conversion/Supplementary_Proofread/figure_sup/real_fake_dP.png]

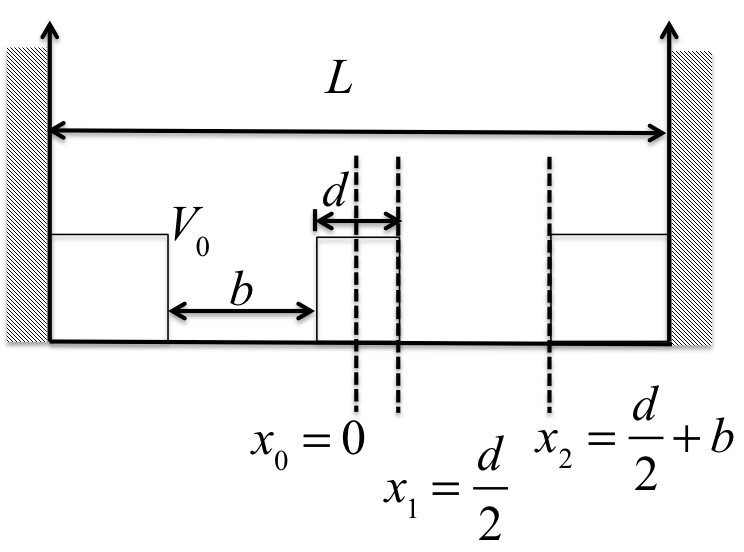

Supplement: Supplementary file 1 [file nanomaterials-12-03599-s001.zip › nanomaterials-1962552-supplementary-conversion/Supplementary_Proofread/figure_sup/two_well_1D.png]

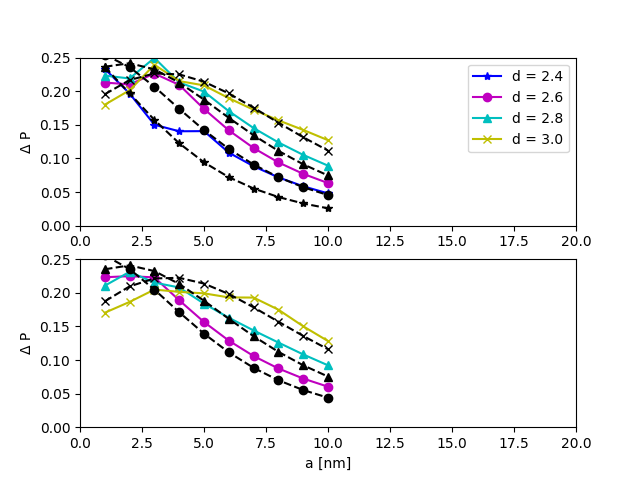

Supplement: Supplementary file 1 [file nanomaterials-12-03599-s001.zip › nanomaterials-1962552-supplementary-conversion/Supplementary_Proofread/figure_sup/V0d6_b2d0_b2d2.png]

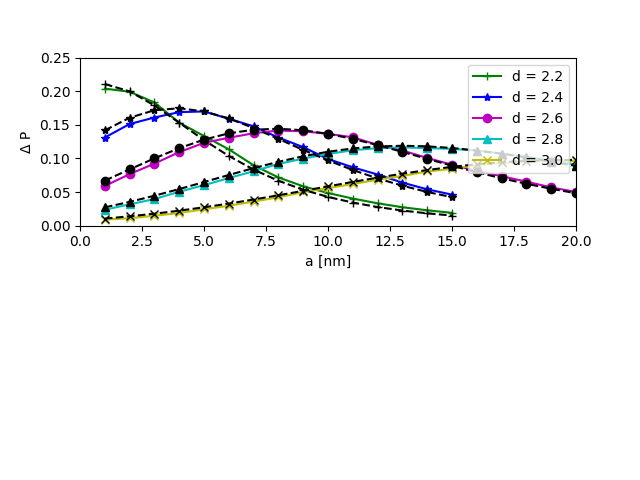

Supplement: Supplementary file 1 [file nanomaterials-12-03599-s001.zip › nanomaterials-1962552-supplementary-conversion/Supplementary_Proofread/figure_sup/V1d5_b1d8.png]
